# H-Y Antigen Incompatibility Not Associated with Adverse Immunologic Graft Outcomes: Deceased Donor Pair Analysis of the OPTN Database

**DOI:** 10.1155/2011/148457

**Published:** 2011-10-15

**Authors:** Douglas Scott Keith, James T. Patrie

**Affiliations:** ^1^Department of Medicine, Division of Nephrology, University of Virginia Medical Center, Charlottesville, VA 22908-0133, USA; ^2^Department of Biostatistics and Epidemiology, University of Virginia, P.O. Box 800717, Charlottesville, VA 22908, USA

## Abstract

*Background*. H-Y antigen incompatibility adversely impacts bone marrow transplants however, the relevance of these antigens in kidney transplantation is uncertain. Three previous retrospective studies of kidney transplant databases have produced conflicting results. *Methods*. This study analyzed the Organ Procurement and Transplantation Network database between 1997 and 2009 using male deceased donor kidney transplant pairs in which the recipient genders were discordant. Death censored graft survival at six months, five, and ten years, treated acute rejection at six months and one year, and rates of graft failure by cause were the primary endpoints analyzed. *Results*. Death censored graft survival at six months was significantly worse for female recipients. Analysis of the causes of graft failure at six months revealed that the difference in death censored graft survival was due primarily to nonimmunologic graft failures. The adjusted and unadjusted death censored graft survivals at five and ten years were similar between the two genders as were the rates of immunologic graft failure. No difference in the rates of treated acute rejection at six months and one year was seen between the two genders. *Conclusions*. Male donor to female recipient discordance had no discernable effect on immunologically mediated kidney graft outcomes in the era of modern immunosuppression.

## 1. Introduction

Identifying candidate minor histocompatibility antigens that impact outcomes after transplantation is difficult, and antigens that have been identified often have disparate effects depending on the organ or tissue transplanted. One minor histocompatibility antigen system that has been identified is the H-Y antigen system. The H-Y antigen system is produced by a series of genes found on the Y chromosome of males which produce proteins with distinct amino acid sequences from their X chromosome homologs and have significant tissue expression. Seven genes have been identified on the Y chromosome that posses these characteristics, and these antigens constitute the H-Y antigen system [1–4]. Organs or tissues from males transplanted into females are potentially exposed to these “nonself” antigens which could produce an alloimmune response. The impact of the H-Y antigen system has been best demonstrated in bone marrow transplants where it has been shown that male bone marrow transplant recipients of marrow transplants from female donors have a higher rate of graft versus host disease, while female recipients of male bone marrow have a higher rate of graft failure than male to male or female to female bone marrow transplants [5–11]. 

The evidence for an effect of the H-Y antigen system in renal transplantation is less certain. The expression of these antigens on the surface of human kidney tissue is not well studied, so it is not clear if target H-Y alloantigens are expressed on kidney tissue. Antibody formation to H-Y antigens has been demonstrated, and in one small study female recipients of male kidneys who had detectable antibodies to H-Y antigens had a higher rate of acute rejection [12]. Registry studies of kidney transplants examining the effect of gender mismatching on outcomes have shown conflicting results. A study of human leukocyte antigen (HLA) identical living donor kidney transplants showed no effect of gender matching on graft survival [13]. A study from an international collaborative transplant registry from 45 countries showed a small but statistically significant poorer death censored graft survival at one and ten years among female recipients of male donor kidneys when compared to all other gender combinations [14]. Finally, an analysis of the Organ Procurement and Transplantation Network (OPTN) database showed a statistically significant difference in death censored graft survival at one year but no difference thereafter [15]. The last two studies used death censored graft survival as a surrogate endpoint for immunologic failure of the graft and used regression analysis to adjust for the differences in the donor and recipient populations, and none of the studies examined acute rejection as an endpoint. 

Since donor quality is an important factor determining both short- and long-term outcomes in kidney transplantation, and the covariates used in modeling do not always capture all the differences, a paired cohort analysis of the OPTN database was undertaken in order to better analyze the impact of the donor recipient gender discordance on kidney transplant outcomes. Because death censored graft failure as an endpoint includes graft failures due to both immunologic and nonimmunologic causes, this paired study includes a more detailed analysis of the causes of graft failure and also an analysis of rejection rates reported to the registry. The primary graft outcomes analyzed were death censored graft survival at six months, five years, and ten years, treated acute rejection rate at six months and one year, and rates of graft failure at six months and from six months onward by putative etiology coded in the database. 

## 2. Patients and Methods

All male deceased donor kidneys from January 1, 1997 to November 27, 2009 in whom the recipient of each pair of kidneys was discordant for recipient gender, were selected from the OPTN database [16]. Only recipient pairs of solitary kidney transplants were included. Pairs in whom one recipient received a multiorgan transplant were excluded. Also pairs in whom survival data was absent on one or both cases were also excluded. Grafts whose cause of failure was primary nonfunction were included in the analysis. No other variables were used to exclude candidates. The donor pair study design was chosen because it controls for donor quality within each pair. This era of transplantation was chosen because the use of the combination of a calcineurin inhibitor and mycophenolic acid type antimetabolites (i.e., modern maintenance immunosuppression) was high. The primary graft outcomes analyzed were death censored graft survival at six months, five years and ten years, treated acute rejection rate at six months and one year, and rates of graft failure at six months and from six months onward by putative etiology coded in the database. 

The etiology of graft failure was determined from two data fields. One was the numerically coded cause of graft failure which included the following categories: hyperacute rejection, acute rejection, primary nonfunction, thrombosis, infection, surgical complication, urologic complication, recurrent disease, chronic rejection, or other. The second field used was a write-in field. For those graft failures coded as other, the write-in field was queried, and based on the documentation two additional causes were included: noncompliance and polyoma-virus-associated nephropathy. Write-in causes of graft failure not included in the list were coded as other. Also, if the query matched one of the numerically coded groups, the cause of graft loss was changed to that category. Graft losses without coding in either field were considered unknown. Recipients who died during followup and whose graft survival time was equal to their patient survival time were classified as death with graft function.

Cox proportional hazard ratio survival analysis was carried out to determine the independent association of graft failure with donor recipient gender match (male to male and male to female). The following covariates were included in the model : recipient age, race/ethnicity (Caucasian, African American, Hispanic, and other), and body mass index (BMI), donor: recipient cytomegalovirus (CMV) serologic status (D+/R−, D+/R+, D−/R+, D−/R−, or unknown) delayed graft function (DGF) (defined as dialysis in the first week after transplant), duration of dialysis prior to transplant, history of previous transplant, cold ischemia time, human leukocyte antigen mismatch, type of induction (lymphocyte depletion, Interleukin-2 receptor blocker, or other/none), maintenance immunosuppression at discharge, and panel reactive antibodies (PRA). 

Statistical significance for categorical variables was determined using the Pearson's chi-squared test and Fisher's exact test. ANOVA testing was used to determine statistical significance between scalar variables. Log rank testing was performed to determine statistical significance for Kaplan Meier survival analysis. Cox analysis included correlation due to the match pairs using the robust sandwich estimate of the covariance matrix specifying the linked recipient-donor pairs [17]. A probability of type 1 error (alpha) = 0.05 two sided was considered to be the threshold of statistical significance. With the exception of the Cox analysis using the robust sandwich estimate of covariance matrix which required the use of Spotfire S + 8.1 (TIBCO Inc., Palo Alto, CA), statistical analysis was performed using SPSS software (version 17.0 for Windows, SPSS, Inc., Chicago, Ill, USA).

## 3. Results

Between January 1, 1997 and November 27, 2009, 12,479 male donors were identified in which both kidneys were transplanted as single kidney transplants in two recipients who were discordant for gender. The characteristics of the pairs are shown in Table 1. Female recipients were slightly younger and more likely to be of minority race or ethnicity, preemptively transplanted, have a PRA greater than 79%, receive lymphocyte depleting agent for induction, and be on tacrolimus and mycophenolic acid derivative for maintenance immunosuppression. Male recipients were more likely to have diabetes mellitus or hypertension as a cause of ESRD, a high-risk CMV serology combination (D+/R−), and delayed graft function. BMI, HLA mismatch, retransplantation, cold ischemia time, and maintenance steroid use were comparable between the two recipient genders. 60.1% of female recipients had at least one pregnancy prior to their transplant.

**Figure 1:**
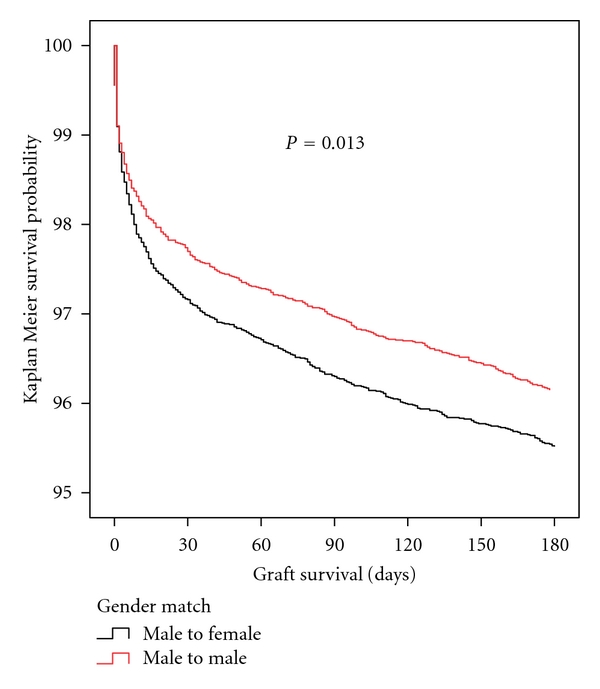
Kaplan Meier plot of death censored graft survival in the first 180 days after kidney transplantation Black line is female recipients of male donor kidneys; red line is male recipients of male donor kidneys. Note that the *y*-axis of survival analysis begins at 100% and ends at 95%. The difference in survival occurs in the first 30 days, and thereafter the slopes of the survival curves are nearly identical.

Figure 1 shows the Kaplan Meier death censored graft survival in the first 180 days after transplant based on gender match. Female recipients of male donors had significantly worse death censored graft failure at 180 days after transplant (log rank *P* = 0.013). The survival curves show that the decrement in survival occurs in the first 30 days. The slopes of the curves after 30 days are nearly identical. Adjusting for the differences in the two populations, female recipients of male kidney had a statistically significant worse outcome when compared to their paired male recipient (Hazard Ratio 1.28 95% C.I. 1.12–1.46). 

Table 2 shows the frequency of graft failure by cause in the first 180 days after transplant. Although hyperacute graft failures were more common in female recipients than male recipients, the main cause of the difference in graft failures seen among female recipients was a higher incidence of graft thrombosis and other specified nonimmunologic causes of graft failure. Since graft failure can be multifactorial, contributory factors were queried for in the dataset. Only 189 cases out of the 1003 cases of graft loss in the first 180 days had a contributory factor listed in the dataset. Considering all immunologic causes both primary and contributory did not change the results of the analysis of graft failures.

Table 3 shows the rate of treated acute rejection at six months and one year in the two recipient genders. 28% and 35% of cases, respectively, lacked coding for treated acute rejection at six months and one year. For each time point, the percentage of missing data for acute rejection was similar between the two genders. That caveat aside the female recipients of male kidneys had a similar rejection rate to male recipients of male kidneys at both time points. 

**Figure 2:**
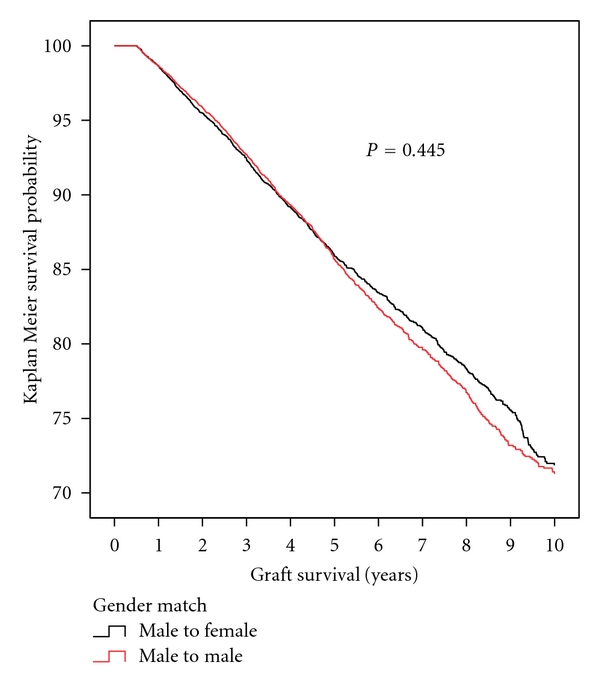
Kaplan Meier plot of death censored graft survival between six months and ten years post kidney transplantation. Black line is female recipients of male donor kidneys and the red line is male recipients of male donor kidneys. Note *y*-axis begins at 100% and ends at 70%.

Figure 2 shows the Kaplan Meier death censored graft survival between six months and ten years. The survival curves were nearly identical with a slight survival advantage seen after five years for female recipients of male kidneys which was not statistically significant. Adjusted hazard ratios for death censored graft survival between six months and five and ten years after transplant were 1.01 (95% CI 0.93–1.11) and 0.98 (95% CI 0.91–1.06) at five and ten years, respectively. The rates of graft loss due to acute and chronic rejection were not different between the two recipient genders. 

## 4. Discussion

This analysis suggests that the male donor to female recipient gender discordance does not have a significant impact on immunologic graft outcomes in the current era of immunosuppression. Our analysis is consistent with the finding from the previous analysis of the OPTN database by Kim and Gill that short-term graft outcomes were worse among female recipients of male kidneys when compared to male recipients while the long-term graft outcomes were not. Our analysis goes further to show that the difference in short-term outcomes occurs very early after transplant and is driven not by immunologically mediated graft failures but by a higher rate of graft thrombosis and graft failure due to other nonimmunologic causes. Similarly, our analysis did not show a difference in the rates in early acute rejection or late graft failures due to acute or chronic rejection consistent with the conclusion that for the vast majority of female recipients of male kidneys receiving modern immunosuppression the H-Y antigen incompatibility did not increase the risk of rejection or graft failure. 

The strength of this study is that it better controls for donor quality and assessed not only death censored graft survival but also the rates of rejection and graft failure by cause. The study has a number of caveats regarding the conclusions. First, the study is limited by the accuracy of the registry data regarding the coding of the cause of graft failure and episodes of rejection. Over 95% of case did have a specified code for cause of graft failure, and the number of cases coded as unknown was similar between the two groups. Determining causality of graft failure can be difficult and depends on medical sophistication of the person entering the data. That being said, there is no reason to believe that the gender of the recipient would bias the presumptive cause of graft failure entered even if the cause of graft failure was incorrect. Second, 28% and 35% of cases, respectively, lacked coding for treated acute rejection at six months and one year. For each time point, the percentage of missing data for acute rejection was similar between the two genders. Although the rates of rejection were similar in the patients in whom coding was present, it is possible that the rejection rates could be different if the missing data were present, and as a result the strength of the conclusion from the acute rejection data must be interpreted in that context. On the other hand, the data from the acute rejection rates is concordant with the findings regarding the rate of immunologic graft loss in this population. 

This study also demonstrates the shortcomings of using death censored graft failure as a surrogate marker for immunologic failure of the graft. Short-term differences in outcomes previously demonstrated in the analysis of the OPTN database appear by our analysis to be due to nonimmunologic graft failures, mainly due to vascular thrombosis and other nonimmunologic causes of renal failure like acute tubular necrosis due to sepsis. In fact, in the present era of immunosuppression, early graft failure in the first six months due to rejection is a rare event occurring in approximately 1% of transplants in this analysis. In contrast, death with functioning graft occurred in approximately 3% of recipients and vascular thrombosis or primary nonfunction in approximately 1%, respectively. The finding of a higher rate of thrombosis among female recipients was unexpected but is biologically plausible given that estrogen is a prothrombotic hormone and females are more likely to have conditions such as antiphospholipid syndrome or lupus erythematous which are associated coagulation abnormalities that increase the risk of thrombosis.

Why H-Y incompatibility did not affect outcomes remains speculative. There are a number of possibilities why one tissue or organ may be more or less susceptible to immunologic attack. One potential explanation for the lack of effect is that H-Y antigen expression on human kidney tissue is absent or weak. There are no published studies of H-Y antigen expression on human kidney tissue. Second, even if the H-Y antigens are expressed on kidneys, the use of potent immunosuppression may prevent kidney rejection in the majority of cases limiting its impact on outcomes. Finally, it is possible that sensitization through previous exposure to the H-Y antigens by pregnancy or previous transplant is necessary before an effect would be seen. One study indicated that the presence of antibodies to H-Y antigens predicted a higher rate of rejection [12]. In our study, 67% of female recipients had a potential sensitizing event via previous organ transplant or pregnancy, the majority being previous pregnancy. Although the gender of the previous donors or fetuses was not know, for each female recipient who had a potential exposure to the H-Y antigens, the probability of exposure can be determined by one minus 0.5 to the power of the number of exposures where 0.5 represents the probability of being exposed to female fetus or donor kidney. Knowing the distribution of the number exposures in the population, one can approximate the overall exposure in the female population that was studied. In this study cohort, approximately 52% of the females would be predicted to have had an exposure either through pregnancy or previous transplant to H-Y antigens. Obviously, exposure to an antigen does not always cause sensitization. But this population did have a significant exposure to H-Y antigens, and no effect was seen suggesting that exposure if it has an effect is weak. Also, it is possible that pregnancy, as is the case of HLA sensitization, is a weaker sensitizing event than exposure through transplantation. Most of the exposures in our population would have been predicted to be due to pregnancy rather than previous transplant potentially attenuating the effect.

In summary, this analysis of the OPTN database indicates that the male donor to female recipient gender discordance has minimal if any impact on immunologic kidney graft outcomes in the modern era of immunosuppression. Female recipients of male donor kidneys had neither an increased rates of acute rejection in the first year after transplant nor increased risk of graft failure related to rejection.

## Figures and Tables

**Table 1:** Patient characteristics.

	Donor male: recipient male (*N* = 12,479)	Donor male: recipient female (*N* = 12,479)	*P* value
Recipient characteristics			
Mean age	48.2 ± 14.9	47.8 ± 15.1	0.05
Race/ethnicity			0.061
Caucasian	50.4%	49.2%	
African American	29.8%	30.4%	
Hispanic	13.6%	13.6%	
Other	6.2%	6.9%	
Mean BMI	27.2 ± 5.2	27.3 ± 6.1	0.13
Cause of endstage renal disease			<0.001
Diabetes Mellitus	24.0%	21.8%	
Glomerulonephritis	16.8%	16.7%	
Hypertension	22.3%	17.2%	
Other/unknown	29.7%	34.9%	
Polycystic kidney disease	7.3%	9.3%	
Duration of dialysis prior to transplant			0.021
Preemptive	8.8%	10.1%	
<1 Year	10.1%	9.7%	
1 to 2 Years	15.8%	15.9%	
2 to 3 Years	15.2%	15.0%	
3 to 4 Years	13.7%	13.4%	
4 to 5 Years	10.4%	9.6%	
More than 5 Years	20.8%	20.9%	
On dialysis at transplant duration unknown	4.8%	4.9%	
Dialysis status not recorded	.4%	.5%	
Previous transplant	13.2%	13.0%	0.69
Previous pregnancy			
Yes	NA	60.1%	
No	NA	25.0%	
Not recorded	NA	14.9%	
PRA			<0.001
0%	73.9%	62.3%	
1–79%	23.2%	30.7%	
80–100%	2.9%	7.0%	
Donor recipient CMV serology			<0.001
Donor +/recipient −	15.6%	12.1%	
Donor + or −/recipient +	51.0%	57.2%	
Donor −/recipient −	7.3%	5.6%	
Missing donor or recipient serology	26.0%	25.1%	
Mean HLA mismatch	3.58 ± 1.86	3.57 ± 1.87	0.56
Cold ischemia time			0.22
0–12 Hours	21.7%	20.7%	
12–24 Hours	48.7%	48.9%	
24–36 Hours	17.1%	17.6%	
>36 Hours	2.9%	2.8%	
Not recorded	9.6%	10.1%	
Delayed graft function			<0.001
Yes	27.0%	21.9%	
No	72.8%	77.9%	
Not recorded	0.2%	0.2%	
Induction therapy			<0.001
Lymphocyte depleting antibody	40.8%	43.3%	
Interleukin 2 receptor antagonist	26.1%	24.3%	
Other/None	33.1%	32.4%	
Discharge maintenance immunosuppression			<0.001
Tacrolimus: MPA Agent	53.4%	54.9%	
Cyclosporine: MPA Agent	22.8%	21.3%	
Tacrolimus or Cyclosporine: azathioprine	3.5%	3.8%	
mTOR any combination	9.0%	8.1%	
Tacrolimus or cyclosporine alone	5.1%	4.9%	
Other combination	6.2%	7.0%	
Discharge maintenance steriods			0.82
No	13.1%	12.9%	
Yes	81.2%	81.5%	
Not specified	5.7%	5.6%	

**Table 2:** Causes of graft loss not due death with function in the first 180 days after transplant.

Variable	Male (*n* = 12479)	Female (*n* = 12479)	Fisher's exact test *P* value
Hyperacute rejection	4 (0.03)	15 (0.12)	0.019
Acute rejection	108 (0.86)	109 (0.87)	1.000
Primary nonfunction	117 (0.93)	118 (0.96)	1.000
Thrombosis	105 (0.84)	149 (1.19)	0.007
Infection	27 (0.21)	21 (0.17)	0.470
Surgical complication	7 (0.06)	8 (0.06)	1.000
Urologic complication	8 (0.06)	4 (0.03)	0.388
Recurrent disease	12 (0.10)	22 (0.18)	0.121
Chronic rejection	31 (0.25)	36 (0.29)	0.625
BK nephropathy	1 (0.01)	2 (0.02)	1.000
Noncompliance	3 (0.02)	1 (0.01)	0.625
Other	26 (0.20)	52 (0.42)	0.004
Unknown	31 (0.25)	23 (0.18)	0.340

**Table 3:** Rate of treated acute rejection at six months and one year by donor recipient gender match.

	Male donor to female recipient	Male donor to male recipient	*P* value
Treated acute rejection in first six months	12.6% (*N* = 8995)	13.1% (*N* = 9005)	0.19
Treated acute rejection in first year	14.8% (*N* = 8124)	15.4% (*N* = 8114)	0.59

Note: Number of cases with data for treated acute rejection for each time interval and gender match are noted in the parentheses in each cell. 28% and 35% of cases in the analysis had missing data for treated acute rejection at six months and one year, respectively. The number of cases with missing data between the two gender match groups was not significantly different.
